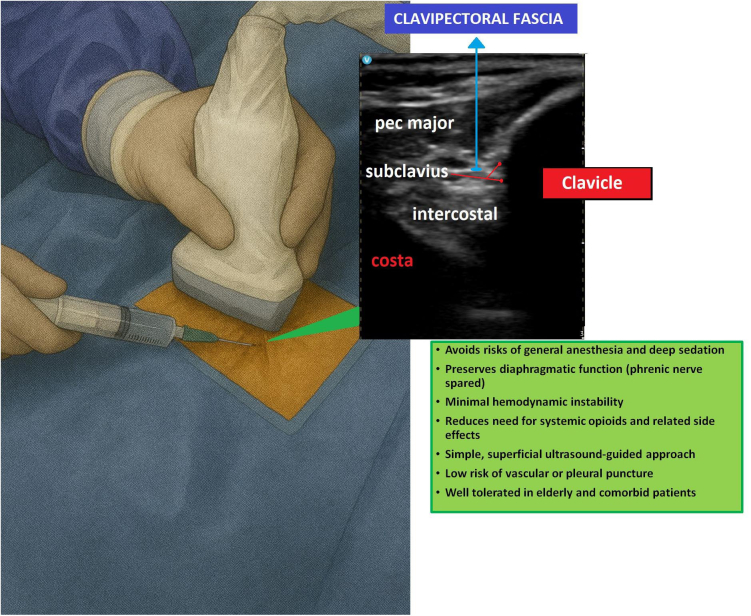# Expanding the role of the clavipectoral fascial plane block: Successful dual-chamber pacemaker implantation with left bundle branch area pacing in an elderly patient

**DOI:** 10.1016/j.hrcr.2025.11.002

**Published:** 2025-11-08

**Authors:** Yakup Yunus Yamanturk, Omer Alkan, Basar Candemir

**Affiliations:** 1 Cardiology Clinic, Artvin State Hospital, Artvin, Turkey; 2 Department of Cardiology, Ankara University, Ankara, Turkey

**Keywords:** Clavipectoral fascial plane block, Elderly patient, Left bundle branch area pacing, Permanent pacemaker implantation, Perioperative analgesia, Regional techniques

**Key Teaching Points**

• The clavipectoral fascial plane block (CFPB) is a simple, ultrasound-guided technique that can provide effective analgesia for pacemaker or defibrillator implantation, reducing the need for opioids and sedatives in elderly patients with comorbidities.
• The block is performed by depositing a local anesthetic agent (15–20 mL of 0.25%–0.5% bupivacaine or ropivacaine, which is approximately equivalent in analgesic potency to 400 mg of prilocaine), between the pectoralis major muscle and the clavipectoral fascia, achieving hydrodissection and spread along the fascial plane.
• The analgesic effect is achieved by blocking small sensory branches, including supraclavicular, lateral pectoral, subclavian, and other minor nerves traversing the clavipectoral fascia, while minimizing the risk of significant motor blockade.
• Safety considerations include avoiding phrenic nerve palsy (more likely with medial/deep injection), vascular puncture (subclavian vessels), and pneumothorax; ultrasound guidance significantly reduces these risks.
• The CFPB offers a feasible and reproducible alternative when anesthesiologist support is limited, making it an attractive adjunct for electrophysiology device implantation.

## Introduction

Permanent pacemaker (PPM) implantation is usually performed under local infiltration anesthesia, occasionally supplemented with mild sedation. Although this approach is sufficient in most cases, patient-related factors such as advanced age, severe anxiety, delirium, claustrophobia, or comorbid conditions may limit tolerability of the procedure and increase the need for sedatives or opioids. Furthermore, discomfort associated with creating the prepectoral pocket highlights the need for more effective and well-tolerated analgesic techniques.

In recent years, ultrasound-guided fascial plane blocks have emerged as valuable alternatives in chest wall surgery. Among these, pectoral nerve blocks (PECS blocks) have been reported to provide adequate intraoperative comfort and up to 24 hours of postoperative analgesia during cardiac implantable electronic device (CIED) procedures. However, these techniques may still produce some degree of motor involvement.^1^, ^2^, ^3^

The clavipectoral fascial plane block (CFPB), initially described in clavicular surgery, involves the injection of a local anesthetic agent into the plane between the pectoralis major muscle and the clavipectoral fascia. By blocking terminal branches traversing this plane, it provides effective analgesia of the anterior chest wall while preserving major motor nerves and reducing the risk of hemidiaphragmatic paralysis compared with interscalene approaches.^4^, ^5^, ^6^

This case report describes a patient from our center, representing the first experience of dual-chamber pacemaker implantation using left bundle branch area pacing (LBBAP) performed under the CFPB.

## Case report

**Figure 1:**
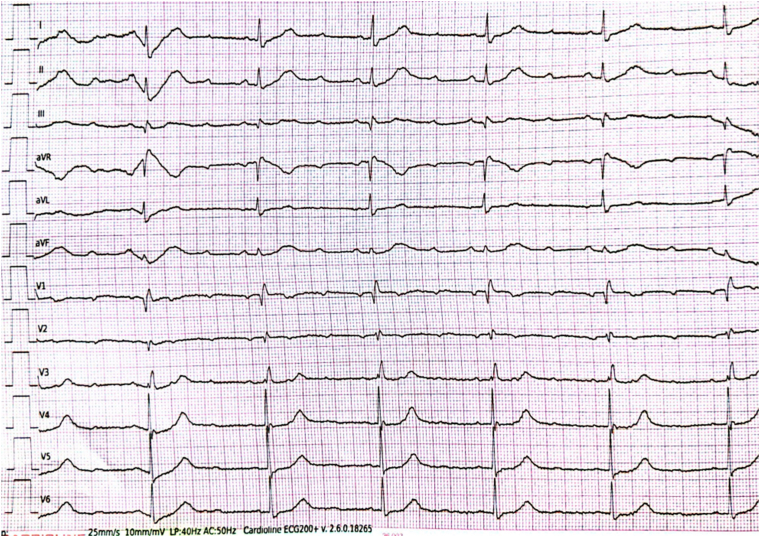
Patient’s admission electrocardiogram that reveals complete atrioventricular block.

An 82-year-old woman with a history of hypertension, atherosclerotic heart disease, paroxysmal atrial fibrillation, and a prior ischemic cerebrovascular accident was admitted to the emergency department with syncope. The admission electrocardiogram revealed complete atrioventricular block, and the patient was urgently transferred to the catheterization laboratory (Figure 1). Her history included verapamil use. A temporary pacemaker was implanted, followed by coronary angiography.

Coronary angiography revealed severe stenosis in the mid–right coronary artery (RCA) and in the distal circumflex artery. As the RCA was dominant, percutaneous coronary intervention was performed on the RCA, whereas implantation of a PPM was deferred for at least 5 days to allow adequate verapamil washout. During this interval, the baseline right bundle branch block pattern on electrocardiography evolved to a narrow QRS complex; however, complete atrioventricular block persisted. Upon deactivation of the temporary pacemaker, the intrinsic ventricular rate fell to 30 beats/min, thereby warranting PPM implantation.

**Figure 2:**
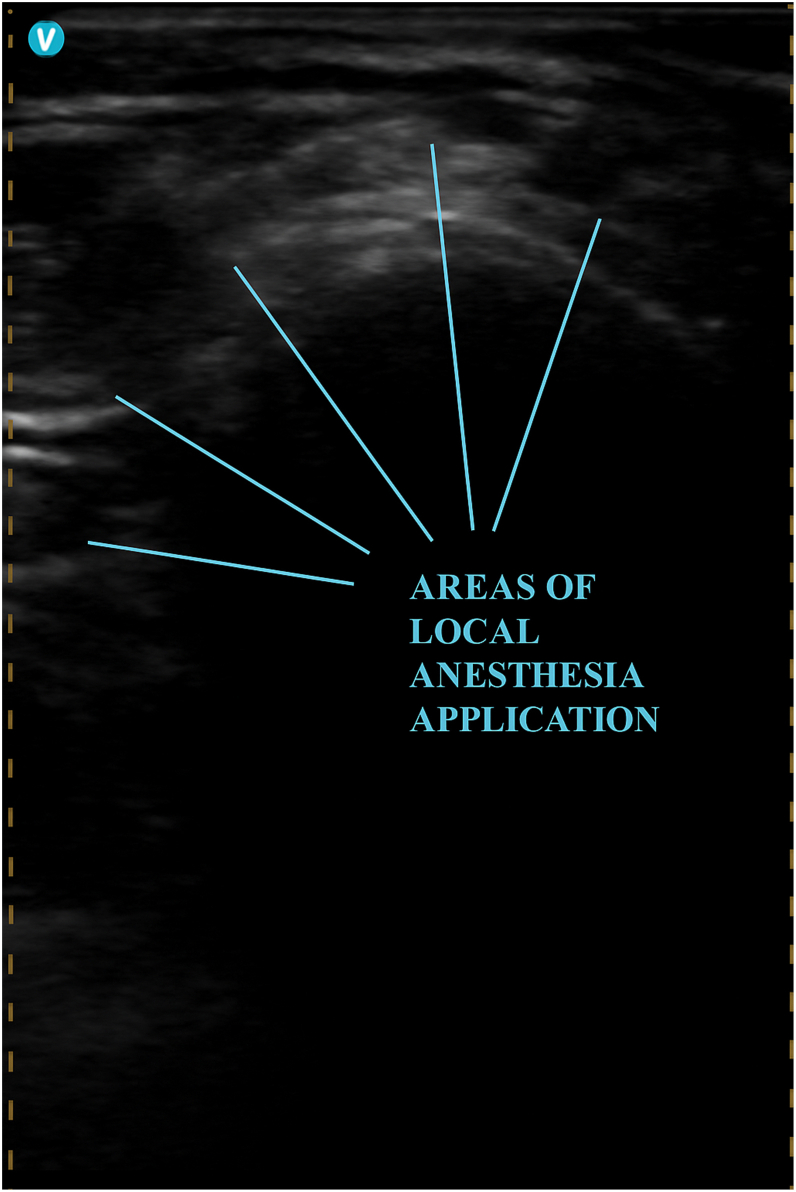
Areas where local anesthetic agents are administered after the clavipectoral fascial plane block.

**Figure 3:**
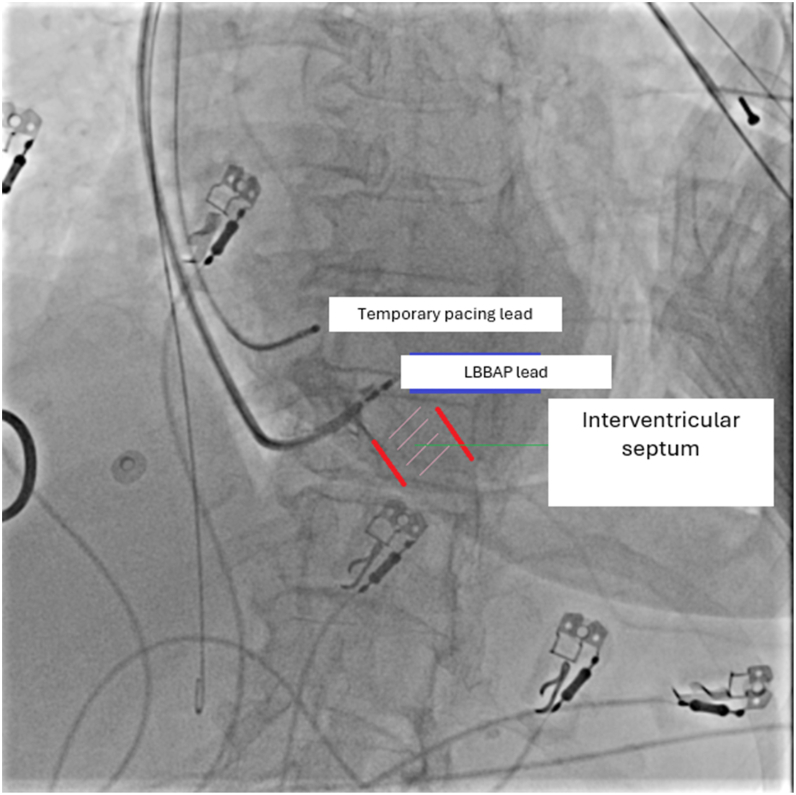
Cineangiographic still image obtained during left bundle branch area pacing lead implantation in the left anterior oblique projection. The lead’s orientation toward the interventricular septum and the intended pacing site are demonstrated (*Red lines* indicate the RV surface and LV surface of the interventricular septum).

**Figure 4:**
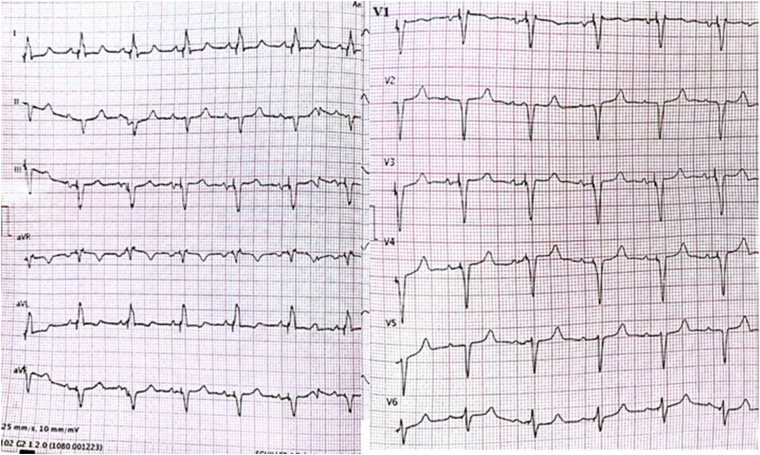
Postprocedural electrocardiogram. The criteria defined in the European Heart Rhythm Association left bundle branch area pacing consensus report are fulfilled.

**Figure 5:**
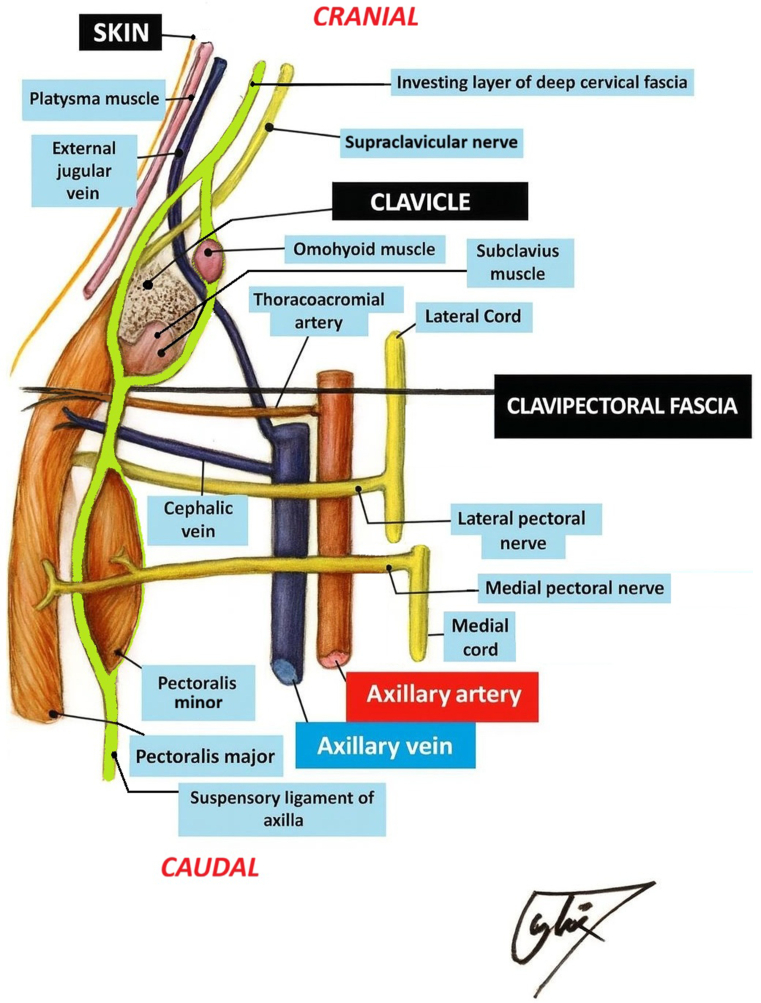
Illustration of the clavipectoral fascia and adjacent anatomical structures. The schematic demonstrates the relationship of the clavipectoral fascia with the pectoralis major and minor muscles, the subclavius muscle, and neighboring neurovascular elements, including the supraclavicular, lateral pectoral, and medial pectoral nerves.

**Figure 6:**
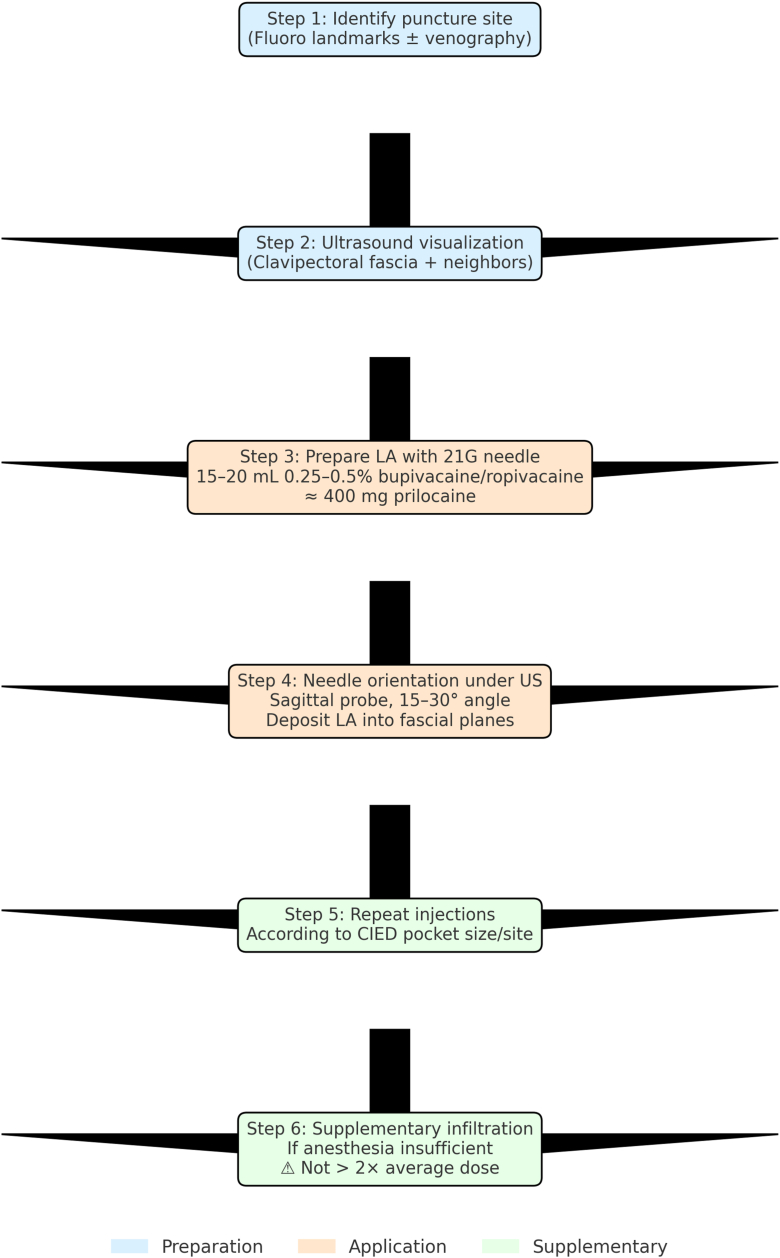
Step-by-step clavipectoral fascial plane block for cardiac implantable electronic device (CIED) implantation. LA = local anesthetic; US = ultrasound.

A dual-chamber Biotronik device (Biotronic Amvia Sky DR-T) with LBBAP capability was implanted via the left axillary vein under the ultrasound-guided CFPB. For the block, a total of 400 mg of prilocaine was administered (Figure 2, Figure 3, Figure 4, Figure 5, Figure 6). The patient remained comfortable, cooperative, and hemodynamically stable throughout the procedure, with no need for additional analgesia. Neither transient nor permanent motor block affecting the surgical field was observed.

In the early postoperative period, the patient reported minimal discomfort, with a maximum Numerical Rating Scale pain score of 1. No methemoglobinemia or pacemaker-related complications occurred. She had an uneventful recovery and was discharged in good condition.

## Discussion

Local infiltration anesthesia is the most commonly used technique to provide analgesia during PPM implantation. However, advanced age, comorbid cardiovascular or neurological disease, anxiety, and drug intolerance may necessitate the use of sedatives or opioids during the procedure. This increases the risk of hemodynamic instability and respiratory depression, especially in patients with limited cardiac reserve.

Ultrasound-guided fascial plane blocks, recently described as minimally invasive and safe techniques, have attracted attention in this patient group. PECS blocks have been successfully applied in CIED implantation procedures, improving intraoperative comfort and prolonging postoperative analgesia. Nevertheless, reports exist of unwanted effects such as motor nerve involvement, and both PECS I–II and interscalene blocks have been associated with cases of motor dysfunction, including inadvertent phrenic nerve paralysis.^2^^,^^3^ The clavipectoral fascia is traversed by the supraclavicular nerve (purely sensory) as well as the lateral and medial pectoral nerves (primarily motor with minor sensory components). The CFPB relies on the spread of the local anesthetic agent within this fascial compartment rather than direct perineural injection, which makes the risk of permanent injury to these nerves exceedingly low. Reported complications are generally limited to transient paresthesia or local anesthetic systemic toxicity. Taken together, although motor involvement and phrenic nerve paralysis have been reported with PECS and interscalene block techniques, the CFPB appears to preserve motor function and minimize the risk of diaphragmatic paralysis, making it the safest and least injurious option among these approaches. The CFPB, first introduced for clavicular surgery, has subsequently been adopted in other chest wall interventions. By targeting the terminal branches innervating the anterior chest wall through injection between the pectoralis major muscle and the clavipectoral fascia, it provides sufficient analgesia while preserving motor nerves and minimizing the risk of diaphragmatic paralysis.^5^, ^6^, ^7^, ^8^ The literature includes a limited number of case reports describing the successful use of the CFPB in PPM or implantable cardioverter-defibrillator implantation.^9^^,^^10^

In our own experience, we have gradually adopted the CFPB as the routine analgesic approach for all CIED implantation procedures, including cardiac resynchronization therapy with defibrillator, implantable cardioverter-defibrillator, and pacemaker implantation procedures with LBBAP. Together with one of our colleagues, we have applied this technique in approximately 20 patients, most of whom were older than 65 years. The total prilocaine dose per case has ranged between 200 and 600 mg, adjusted according to body surface area and device size (Figure 6). Importantly, we have not encountered serious complications such as methemoglobinemia, systemic toxicity, or clinically relevant motor deficits (eg, phrenic nerve paralysis), which have been reported, although rarely, with other regional techniques such as interscalene block. This observation further supports the relative safety of the CFPB compared with pectoral and interscalene approaches.

Compared with conventional pacemaker implantation, the advantages of the CFPB may be even more pronounced in conduction system pacing, particularly LBBAP. These procedures are technically demanding and often prolonged, where superior patient comfort, immobility, and reduced sedation requirements are of greater importance.

In the present case, the CFPB enabled the completion of PPM implantation with LBBAP in an elderly patient with significant cardiac comorbidities, without the need for supplemental opioids or sedatives. The patient experienced low pain scores and required no additional analgesia in the early postoperative period, supporting the efficacy of this approach (Supplemental Figure 1). Moreover, despite the moderate-to-high total dose of prilocaine, no complications such as methemoglobinemia or local anesthetic systemic toxicity were observed.

This case illustrates that the CFPB may be a valuable alternative for patients at high cardiac risk, with poor tolerance to sedation and high expectations of comfort. Nevertheless, as current evidence is mainly limited to case reports, larger prospective studies are necessary to confirm the safety and efficacy of this technique.

## Conclusion

The CFPB provides a safe, effective, and well-tolerated analgesic option during PPM implantation with LBBAP. It may be particularly beneficial in elderly patients with comorbidities, in whom the avoidance of sedatives and opioids is desirable. However, further prospective randomized trials are required to establish its role in routine clinical practice.

## Disclosures

The authors have no conflicts of interest to disclose.

## Figures and Tables

**Supplementary figure 1:**