# Supplementary material for: Expanding the role of the clavipectoral fascial plane block: Successful dual-chamber pacemaker implantation with left bundle branch area pacing in an elderly patient
Source: HeartRhythm Case Rep. 2025 Nov 8;12(2):219–25. doi: 10.1016/j.hrcr.2025.11.002 (PMC12922523; doi:10.1016/j.hrcr.2025.11.002)
Supplement: Supplementary figure [file mmc1.docx]

**Supplementary figure 1 description.** Summary of the advantages of using the clavipectoral fascial plane block during dual-chamber pacemaker implantation with left bundle branch area pacing (LBBAP)
